# Impact of Prior Treatment History on Recurrence After Complete Response to Atezolizumab Plus Bevacizumab in Unresectable Hepatocellular Carcinoma

**DOI:** 10.1002/cam4.71552

**Published:** 2026-01-26

**Authors:** Tasuku Nakabori, Masaki Kawabata, Kaori Mukai, Hidenari Hongyo, Noboru Maeda, Makiko Urabe, Yugo Kai, Ryoji Takada, Kenji Ikezawa, Hiroshi Wada, Kunihito Gotoh, Kazuyoshi Ohkawa

**Affiliations:** ^1^ Department of Hepatobiliary and Pancreatic Oncology Osaka International Cancer Institute Osaka Japan; ^2^ Department of Diagnostic and Interventional Radiology Osaka International Cancer Institute Osaka Japan; ^3^ Department of Gastroenterological Surgery Osaka International Cancer Institute Osaka Japan

**Keywords:** atezolizumab plus bevacizumab, hepatocellular carcinoma, immunotherapy, recurrence

## Abstract

**Aim:**

Integration of locoregional treatments (LRTs) with atezolizumab plus bevacizumab (atezo/bev) has improved treatment outcomes, enabling an increasing number of patients with unresectable hepatocellular carcinoma (HCC) to achieve a complete response (CR). A comprehensive analysis of recurrence following CR may provide insights into prognosis. This study aimed to identify the factors associated with post‐CR recurrence in unresectable HCC.

**Methods:**

This retrospective study included 15 patients with unresectable HCC who achieved CR with atezo/bev therapy. The incidence and characteristics of post‐CR recurrence were analyzed.

**Results:**

Ten patients achieved CR by combining LRT with atezo/bev, whereas five achieved CR with atezo/bev alone. The post‐CR recurrence rate was 53.3% (recurrence group, *n* = 8; non‐recurrence group, *n* = 7). No significant differences were observed between the recurrence and non‐recurrence groups in HCC treatment history prior to atezo/bev initiation, predictive factors for treatment response, including the neutrophil‐to‐lymphocyte ratio, hepatic functional reserve, and tumor burden at the initiation of atezo/bev, treatment progress, or frequency of atezo/bev maintenance therapy post‐CR. In multivariate analysis, a history of ≥ 2 HCC treatments prior to atezo/bev initiation was independently associated with post‐CR recurrence (hazard ratio, 6.744; 95% confidence interval, 1.189–38.25; *p* = 0.031); conversely, predictive factors for atezo/bev response, treatment progress, and maintenance therapy did not contribute to post‐CR recurrence.

**Conclusions:**

Given the high post‐CR recurrence rate with atezo/bev in unresectable HCC, vigilant surveillance remains essential even after achieving CR, particularly in patients with ≥ 2 prior HCC treatments before atezo/bev initiation.

**Abbreviations**
AFP: alpha‐fetoprotein
ALBI: albumin‐bilirubin
atezo/bev: atezolizumab plus bevacizumab
BCLC: Barcelona Clinic Liver Cancer
CI: confidence interval
CR: complete response
CT: computed tomography
DCP: des‐gamma‐carboxy prothrombin
EOB‐MRI: gadolinium‐ethoxybenzyl diethylenetriamine pentaacetic acid‐enhanced magnetic resonance imaging
HCC: hepatocellular carcinoma
HR: hazard ratio
LRT: locoregional treatment
NLR: neutrophil‐to‐lymphocyte ratio
PD: progressive disease
PR: partial response
RFA: radiofrequency ablation
RFS: recurrence‐free survival
SBRT: stereotactic body radiotherapy
SD: stable disease
TACE: transarterial chemoembolization

## 1 Introduction

Combination immunotherapy, including atezolizumab plus bevacizumab (atezo/bev) and the combination of durvalumab and tremelimumab, has demonstrated superior efficacy in patients with unresectable hepatocellular carcinoma (HCC) compared with sorafenib [1], the previous standard first‐line therapy, in pivotal phase III trials (IMbrave150 [2] and HIMALAYA [3]). Consequently, these regimens have received global approval as first‐line systemic therapies for unresectable HCC [4, 5, 6]. Although atezo/bev offers a relatively high (27.1%–30.8%) and durable therapeutic response, the complete response (CR) rate remains low at only 0%–7.7% [2, 7, 8].

Recent studies have reported the clinical efficacy of combining systemic therapy with multidisciplinary treatments, including surgery, ablation, transcatheter arterial chemoembolization (TACE), and radiotherapy with curative intent, a strategy known as conversion therapy [9, 10]. The addition of locoregional treatment (LRT) has improved treatment outcomes, increasing CR rates to 10.4%–34.5% [11, 12, 13, 14]. This therapeutic approach is now established and has enabled an increasing number of patients with unresectable HCC to achieve CR. Predictive factors associated with therapeutic response to atezo/bev or transition to conversion therapy, including early tumor response, low neutrophil‐to‐lymphocyte ratio (NLR), preserved hepatic functional reserve, and low tumor burden, have been identified [11, 14, 15, 16, 17]. However, recurrence after CR and its contributing factors have not been thoroughly investigated.

To address this gap, this study aimed to analyze the predictive factors for recurrence following CR in patients with unresectable HCC treated with atezo/bev.

## 2 Methods

### 2.1 Study Population

We retrospectively collected clinical data from patients with unresectable HCC who received atezo/bev as first‐ or second‐line systemic therapy at the Osaka International Cancer Institute, Osaka, Japan, between November 1, 2020, and March 31, 2024. HCC was diagnosed according to American Association for the Study of Liver Diseases criteria [18, 19]. Tumor staging was performed using the Barcelona Clinic Liver Cancer (BCLC) staging system [20, 21]. Inclusion and exclusion criteria were applied as previously reported [13]. The atezo/bev therapy initiation date was defined as the beginning of the follow‐up period, which concluded on March 31, 2025.

This study was conducted in accordance with the principles of the Declaration of Helsinki and was approved by the Institutional Review Board for Clinical Research at the Osaka International Cancer Institute (approval number 25007), which waived the requirement for informed consent. An opt out approach was implemented via the hospital website to provide patients with the opportunity to decline participation in the study.

### 2.2 Atezo/Bev Treatment

Patients received atezolizumab (1200 mg) and bevacizumab (15 mg/kg) every 3 weeks. In the event of treatment‐related adverse events, administration of one or both agents was temporarily suspended until symptoms resolved to grade 1 or lower, in accordance with National Cancer Institute Common Terminology Criteria for Adverse Events (version 5.0). Dynamic contrast‐enhanced computed tomography (CT) or gadolinium‐ethoxybenzyl diethylenetriamine pentaacetic acid‐enhanced magnetic resonance imaging (EOB‐MRI) was performed every 6–9 weeks during atezo/bev therapy.

Discontinuation of atezo/bev after achieving CR was primarily determined by meeting the following criteria for at least 6 months: (1) confirmation of CR via contrast‐enhanced CT or EOB‐MRI and (2) normalization of alpha‐fetoprotein (AFP) and *Lens culinaris* agglutinin‐reactive fraction of AFP (AFP‐L3). However, treatment discontinuation was also considered in cases of significant adverse events owing to atezo/bev or at the patient's request.

### 2.3 Combination of LRT

LRT, including radiofrequency ablation (RFA), TACE, surgical resection, or stereotactic body radiotherapy (SBRT), was combined with atezo/bev for two purposes: first, to eradicate residual viable tumors after significant reduction by atezo/bev, thereby achieving CR, known as curative conversion therapy (ABC conversion) [9, 10]; and second, to treat oligo‐drug‐resistant lesions that emerged during atezo/bev therapy to salvage progressive disease (PD) status, followed by continuation of atezo/bev (PD salvage) [13, 22]. The criteria for LRT in ABC conversion or PD salvage were applied as previously described [13, 22].

### 2.4 Evaluation of Therapeutic Response and Hepatic Reserve

Therapeutic responses were assessed using dynamic contrast‐enhanced CT or EOB‐MRI, based on modified Response Evaluation Criteria in Solid Tumors [23]. Responses were categorized as CR, partial response (PR), stable disease (SD), or PD.

Recurrence‐free survival (RFS) was defined as the period between the achievement of CR and the onset of recurrence. Early response was defined as a therapeutic response observed after two cycles of atezo/bev administration.

Hepatic functional reserve was evaluated using the Child–Pugh score, albumin‐bilirubin (ALBI) score, and modified ALBI grade [24, 25].

### 2.5 Surveillance Following CR Achievement

To monitor recurrence, tumor markers were measured every 1–3 months, and imaging assessments, including contrast‐enhanced CT or EOB‐MRI, were performed at 3‐month intervals or when tumor markers were elevated.

### 2.6 Statistical Analysis

Continuous variables are expressed as medians (ranges) and were analyzed using the Mann–Whitney *U* or Wilcoxon signed‐rank tests, as appropriate. Categorical variables are presented as absolute counts and were analyzed using Pearson's chi‐square or Fisher's exact tests, as applicable. RFS was estimated using the Kaplan–Meier method and comparisons were performed using a log‐rank test. Hazard ratios (HRs) and 95% confidence intervals (CIs) were calculated using a multivariate Cox proportional hazards model. Statistical significance was set at *p* < 0.05. All statistical analyses were conducted using EZR (Saitama Medical Center, Jichi Medical University, Saitama, Japan), a graphical user interface for the R Commander software (version 1.61) designed for Windows [26].

## 3 Results

### 3.1 CR Achievement

**FIGURE 1:**
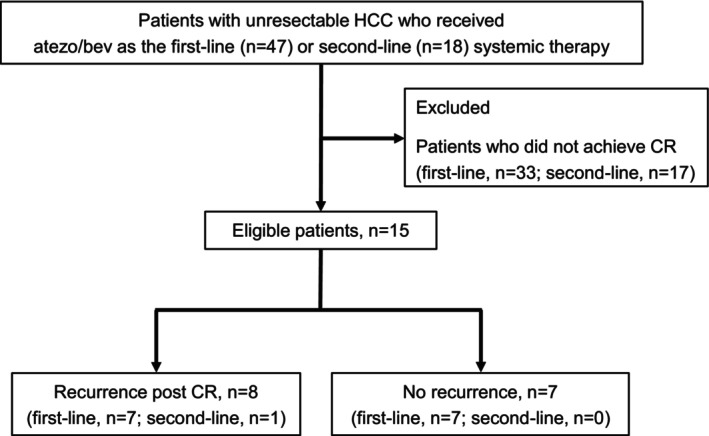
Flowchart of study enrollment. atezo/bev, atezolizumab plus bevacizumab; CR, complete response; HCC, hepatocellular carcinoma.

A total of 65 patients with unresectable HCC who received atezo/bev as first‐line (*n* = 47) or second‐line (*n* = 18) systemic therapy were screened (Figure 1). Among the second‐line patients, first‐line therapies included lenvatinib [27] (*n* = 17) and sorafenib [1] (*n* = 1). Fifty patients were excluded owing to failure to achieve CR with atezo/bev. The remaining 15 patients achieved CR. The overall CR rate among all of the eligible patients was 23.1%. The CR rate in the first‐line group (14/47, 29.8%) was higher than that in the second‐line group (1/18, 5.6%; *p* = 0.049). The median observation periods from atezo/bev initiation and CR achievement were 1124 (range, 419–1617) days and 553 (range, 132–1424) days, respectively. No deaths occurred among the enrolled patients during the observation period (Figure S1A,B). The median duration from atezo/bev therapy initiation to CR was 310 (range, 193–799) days.

### 3.2 Recurrence Post‐CR

**FIGURE 2:**
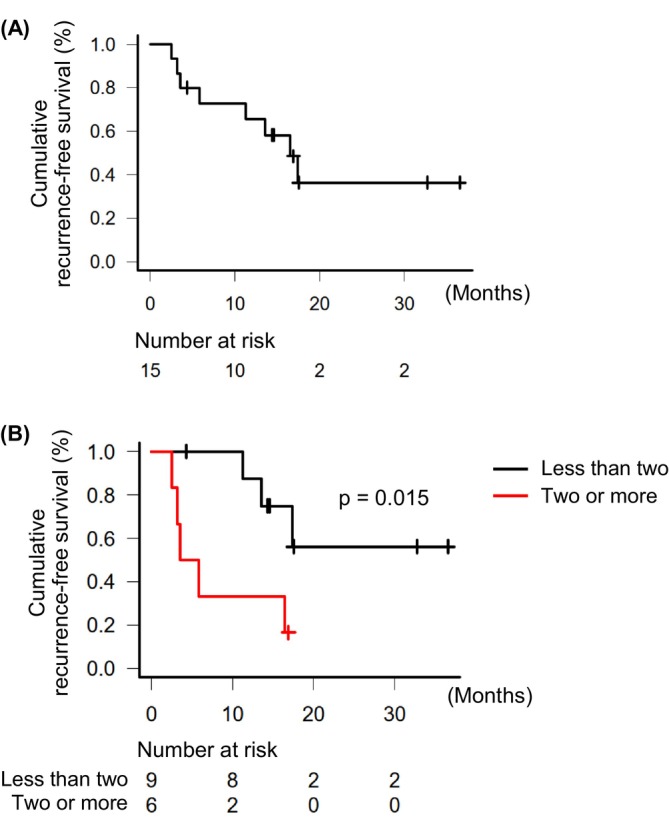
Kaplan–Meier curves for RFS after achieving complete response. (A) Overall RFS in all eligible patients. (B) Comparison of RFS between patients with two or more prior hepatocellular carcinoma treatments and those with fewer than two. RFS, recurrence‐free survival.

Eight patients experienced recurrence post‐CR (recurrence group; Table S1), whereas the remaining seven maintained CR during the observation period (non‐recurrence group). The recurrence rate post‐CR was 53.3%. The median duration from CR to recurrence was 16.5 months (95% CI, 3.5 months–not available) (Figure 2A). The baseline characteristics at atezo/bev therapy initiation and therapeutic efficacy of atezo/bev are presented in Table 1. All patients with BCLC stage B HCC exceeded the up‐to‐seven criteria (substage B2) [21]. Among the nine patients with BCLC stage C HCC, one had portal vein tumor thrombosis and eight had extrahepatic metastases, including lung (*n* = 3), lymph nodes (*n* = 4), bone (*n* = 2), and adrenal glands (*n* = 2); these counts include patients with multiple metastatic sites. Ten patients achieved CR by receiving LRT in combination with atezo/bev, whereas five achieved CR with atezo/bev alone. The details of the combined LRTs administered to these ten patients are summarized in Table S1. Thirteen patients maintained atezo/bev therapy following CR. A comparison between the recurrence and non‐recurrence groups revealed no significant differences regarding age, sex, etiology, hepatic functional reserve, tumor‐related factors (including staging and tumor markers), or a history of HCC treatment such as RFA, TACE, or surgical resection, at the time of atezo/bev initiation. No differences were observed in treatment progress with atezo/bev, including early tumor response and duration from atezo/bev initiation to CR. No significant difference was observed in the frequency of post‐CR atezo/bev maintenance therapy between the two groups. Seven patients in the recurrence group received atezo/bev maintenance therapy post‐CR, of whom five experienced recurrences during maintenance (Table S2). Three recurrences that occurred during atezo/bev maintenance therapy did not exhibit distinct early arterial phase enhancement on CT (Figure S2A). In contrast, two recurrences observed during maintenance with atezolizumab monotherapy, administered owing to adverse events, demonstrated clear early arterial phase enhancement on CT (Figure S2B). No significant differences in tumor marker levels, including AFP (*p* = 0.098), AFP‐L3 (*p* = 1.000), and des‐gamma‐carboxy prothrombin (*p* = 0.205), were observed between the time of CR achievement and that of recurrence (Table S3). All eight recurrences were intrahepatic; no extrahepatic metastatic recurrences were observed. Six patients were within the Milan criteria [28]: five were treated with RFA, and one with SBRT. The remaining two were beyond the Milan criteria: one within the up‐to‐seven criteria (four intrahepatic lesions ≤ 3 cm in diameter) was treated with RFA, based on previous reports demonstrating its efficacy for intermediate‐stage HCC [29, 30]; the other, who was beyond the up‐to‐seven criteria [21] and receiving atezo/bev maintenance therapy, was switched to lenvatinib.

**TABLE 1:** Comparison of baseline characteristics and therapeutic efficacy with atezo/bev therapy between the recurrence and non‐recurrence groups.

Variable	All patients (*n* = 15)	Recurrence (*n* = 8)	Non‐recurrence (*n* = 7)	*p*
Age, years	72 (49–81)	77 (63–81)	66 (49–81)	0.246
Sex, male/female	15/0	8/0	7/0	1.000
Etiology, viral/nonviral	3/12	2/6	1/6	1.000
Child‐Pugh score, 5/6/7	11/3/1	4/3/1	7/0/0	0.128
ALBI score	−2.676 (−3.283 to −1.970)	−2.491 (−3.283 to −1.970)	−2.733 (−3.011 to −2.416)	0.694
Modified ALBI grade, 1/2a/2b	8/4/3	4/1/3	4/3/0	0.204
Platelet count, 10^4^/μL	18.8 (10.1–36.4)	17.1 (12.4–36.4)	18.7 (10.1–23.4)	0.908
NLR	2.634 (1.507–4.463)	3.445 (1.922–4.463)	2.546 (1.507–3.984)	0.189
Serum AFP, ng/mL	5601 (2–55,310)	7 (2–55,310)	5 (2–26,055)	0.907
Serum DCP, mAU/mL	5602 (< 30–37,152)	114 (< 30–5148)	574 (< 30–37,152)	0.182
BCLC stage, B^a^/C	6/9	4/4	2/5	0.608
Macrovascular invasion, −/+	14/1	7/1	7/0	1.000
Extrahepatic metastasis, −/+	7/8	5/3	2/5	0.315
Number of lesions	4 (1–39)	4 (1–30)	5 (3–39)	0.377
Maximum diameter of the tumor, mm	39 (14–162)	27 (18–62)	67 (14–162)	0.132
Primary/recurrent	6/9	2/6	4/3	0.315
Number of times of HCC treatments^b^ prior to atezo/bev initiation	1 (0–13)	2 (0–13)	0 (0–3)	0.118
Treatment line, first/s	14/1	7/1	7/0	1.000
Early tumor response^c^, CR/PR/SD/PD	0/7/8/0	0/3/5/0	0/4/3/0	0.619
Combined LRT, −/+	5/10	1/7	4/3	0.119
Duration from atezo/bev initiation to CR, days	310 (193–799)	407 (193–799)	287 (204–427)	0.397
Maintenance therapy with atezo/bev post‐CR, −/+	2/13	1/7	1/6	1.000

*Note:* Continuous variables are shown as median (range).

Abbreviations: −/+, without/with; AFP, α‐fetoprotein; ALBI score, albumin‐bilirubin score; atezo/bev, atezolizumab plus bevacizumab; BCLC, Barcelona Clinic Liver Cancer; CR, complete response; DCP, des‐γ‐carboxy prothrombin; HCC, hepatocellular carcinoma; LRT, locoregional treatment; NLR, neutrophil‐to‐lymphocyte ratio; PD, progressive disease; PR, partial response; SD, stable disease.

^a^ All patients with BCLC stage B had a tumor burden exceeding the up‐to‐seven criteria.

^b^ HCC treatment includes radiofrequency ablation, transarterial chemoembolization, or surgical resection.

^c^ Early tumor response indicates a therapeutic response observed after two cycles of atezo/bev.

### 3.3 Analysis of Factors Contributing to Post‐CR Recurrence

The factors contributing to post‐CR recurrence were analyzed (Table 2). The number of times of HCC treatments prior to atezo/bev initiation (HR, 1.231; 95% CI, 1.019–1.486; *p* = 0.031), particularly ≥ 2 prior HCC treatments (HR, 6.128; 95% CI, 1.167–32.17; *p* = 0.032), was associated with post‐CR recurrence in univariate analysis. Two factors with low *p*‐values, namely, NLR ≥ 3 and ≥ 2 HCC treatments prior to atezo/bev initiation, were included in the multivariate logistic regression model. Two or more HCC treatments prior to atezo/bev initiation were independently associated with post‐CR recurrence (HR, 6.744; 95% CI, 1.189–38.25; *p* = 0.031).

**TABLE 2:** Factors contributing to recurrence post‐CR.

	Univariate analysis			Multivariate analysis		
	HR	95% CI	*p*	HR	95% CI	*p*
Age, < 75 vs. ≥ 75	2.867	0.517–15.89	0.228			
Etiology, viral vs. nonviral	2.056	0.402–10.51	0.387			
Child‐Pugh score, 5 vs. 6 or 7	2.899	0.718–11.63	0.135			
Modified ALBI grade, 1 vs. 2a or 2b	1.675	0.354–7.937	0.516			
NLR, < 3 vs. ≥ 3	3.292	0.798–13.58	0.099	3.796	0.774–18.62	0.100
Serum AFP, < 400 vs. ≥ 400 ng/mL	0.862	0.168–4.428	0.859			
Serum DCP, < 1000 vs. ≥ 1000 mAU/mL	3.247	0.392–27.03	0.275			
BCLC stage, B vs. C	1.779	0.440–7.194	0.419			
Primary vs. recurrent	1.958	0.384–9.978	0.419			
Number of times of HCC treatments^a^ prior to atezo/bev initiation	1.231	1.019–1.486	**0.031**			
Two or more HCC treatments^a^ prior to atezo/bev initiation, yes vs. no	6.128	1.167–32.17	**0.032**	6.744	1.189–38.25	**0.031**
Number of lesions, < 4 vs. ≥ 4	0.960	0.240–3.847	0.954			
Maximum diameter of the tumor, < 40 vs. ≥ 40 mm	0.369	0.074–1.843	0.225			
Early tumor response^b^, PR vs. SD	2.161	0.509–9.178	0.297			
Combined LRT, yes vs. no	0.202	0.025–1.650	0.135			
Duration from atezo/bev initiation to CR, < 300 vs. ≥ 300 days	1.844	0.435–7.807	0.406			
Maintenance therapy with atezo/bev post‐CR, yes vs. no	1.556	0.190–12.75	0.680			

*Note:* A bolded *p*‐value indicates statistical significance (*p* < 0.05).

Abbreviations: −/+, without/with; AFP, α‐fetoprotein; ALBI score, albumin‐bilirubin score; atezo/bev, atezolizumab plus bevacizumab; BCLC, Barcelona Clinic Liver Cancer; CI, confidence interval; DCP, des‐γ‐carboxy prothrombin; EHM, Extrahepatic metastasis; HCC, hepatocellular carcinoma; HR, hazard ratio; LRT, locoregional treatment; MVI, Macrovascular invasion; NLR, neutrophil‐to‐lymphocyte ratio; PR, partial response; SD, stable disease.

^a^ HCC treatment includes radiofrequency ablation, transarterial chemoembolization, or surgical resection.

^b^ Early tumor response indicates a therapeutic response observed after two cycles of atezo/bev.

### 3.4 Comparison of Recurrence Post‐CR in Patients With Two or More and Less Than Two Prior HCC Treatments Before Atezo/Bev Initiation

The enrolled patients were divided into two groups (‘two or more’ and ‘less than two’), based on the frequency of HCC treatments—such as RFA, TACE, or surgical resection—before atezo/bev initiation, with two as the cutoff. Baseline characteristics at atezo/bev initiation and its therapeutic efficacy are presented in Table S4. No significant differences were observed between the two groups, except for variables related to HCC treatment prior to atezo/bev initiation.

RFS was compared between the two groups. RFS was shorter in the two or more group (median, 4.7 months; 95% CI, 2.5 months–not available) than in the less than two group (median, not available; 95% CI, 11.3 months–not available) (*p* = 0.015) (Figure 2B).

## 4 Discussion

Despite significant advancements in systemic therapies, achieving a CR with systemic therapy alone remains rare in patients with unresectable HCC. However, integrating LRTs with atezo/bev has increased CR rates in this population, and the number of patients achieving CR is expected to grow. A detailed analysis of recurrence following CR may support further prognostic improvements. Therefore, this study aimed to identify factors associated with recurrence after CR in patients with unresectable HCC.

In this study, the CR rate of all the eligible patients was comparable to that reported in previous studies (10.4%–34.5%) [11, 12, 14]. Two‐thirds of the patients received LRTs to achieve a CR. The CR rate of atezo/bev as the first‐line treatment was higher than that as the second‐line treatment, which is consistent with a previous report [31]. More than half of the patients experienced recurrence after achieving CR during a follow‐up period of approximately 1.5 years. This recurrence rate is relatively high compared with that reported in other cancer types among patients who achieved CR with immune checkpoint inhibitors (13.4%–22.5%) [32, 33]. Tumor marker levels at the time of recurrence were not elevated compared with those at the time of CR achievement. These results indicate that close imaging surveillance at 3‐month intervals after achieving CR may facilitate the early detection of recurrence. Regarding risk factors for recurrence post‐CR, a history of ≥ 2 HCC treatments, including RFA, TACE, or surgical resection prior to atezo/bev initiation, was identified. Conversely, previously reported predictive factors for response to atezo/bev or transition to conversion therapy, such as early tumor response to atezo/bev, NLR, hepatic functional reserve, or tumor burden [11, 14, 15, 16, 17], and combined LRT to achieve CR were not associated with recurrence post‐CR. All recurrences were intrahepatic and > 50% were solitary. These findings support the possibility that recurrence after achieving CR with atezo/bev may represent de novo tumor development associated with underlying liver disease, as evidenced by the high frequency of intrahepatic recurrence—a characteristic feature of HCC [34, 35]—rather than the emergence of occult atezo/bev‐resistant lesions.

Maintenance therapy with atezo/bev following CR was not associated with a reduced recurrence risk. More than half of the patients who continued atezo/bev after achieving CR eventually experienced recurrence. Furthermore, at the time of recurrence, more than half of these patients were still receiving maintenance therapy. These findings suggest that atezo/bev maintenance therapy post‐CR may have limited efficacy in preventing recurrence. In contrast, early arterial phase enhancement on CT was attenuated in recurrences during atezo/bev maintenance therapy, whereas it remained evident in those observed during atezolizumab monotherapy maintenance. This phenomenon may be attributable to the anti‐vascular endothelial growth factor effect of bevacizumab, which suppresses vascular endothelial cell proliferation and angiogenesis [36, 37]. Consequently, maintenance therapy with atezo/bev may contribute to suppress microscopic disease or delay the emergence of overt recurrence. Further investigation is warranted to determine the necessity and optimal duration of atezo/bev maintenance therapy after CR.

This study has several limitations. The small sample size, retrospective design, and single‐center setting may have introduced selection and information biases. Large‐scale prospective studies and real‐world investigations across multiple institutions are needed to validate our findings.

In conclusion, the recurrence rate after CR with atezo/bev was relatively high in patients with unresectable HCC. Recurrence was associated with a history of prior HCC treatments before atezo/bev initiation, but not with established predictors of response to atezo/bev. Appropriate surveillance for recurrence remains essential even after achieving a CR, particularly in patients with ≥ 2 prior HCC treatments before initiating atezo/bev.

## Author Contributions

Conceptualization and writing: Tasuku Nakabori. Review and editing: Tasuku Nakabori and Kazuyoshi Ohkawa. Data curation and formal analysis: Tasuku Nakabori and Masaki Kawabata. Methodology: Tasuku Nakabori. Resouces: Tasuku Nakabori, Masaki Kawabata, Kaori Mukai, Hidenari Hongyo, Noboru Maeda, Makiko Urabe, Yugo Kai, Ryoji Takada, Kenji Ikezawa, Hiroshi Wada, Kunihito Gotoh, and Kazuyoshi Ohkawa. All authors have read and approved the final version of the manuscript.

## Funding

The authors have nothing to report.

## Ethics Statement

The protocol for this research project has been approved by a suitably constituted Ethics Committee of the institution and it conforms to the provisions of the Declaration of Helsinki. The Institutional Review Board for Clinical Research at Osaka International Cancer Institute, Approval no. 25007.

## Consent

The Institutional Review Board for Clinical Research of Osaka International Cancer Institute (approval number: 25007) waived the requirement for informed consent. The opt out method was provided to the patients on our hospital's website.

## Conflicts of Interest

The authors declare no conflicts of interest.

## Supporting information


**Figure S1:** cam471552‐sup‐0001‐FigureS1.pptx.

**Figure S2:** cam471552‐sup‐0002‐FigureS2.pptx.

**Table S1:** cam471552‐sup‐0003‐TableS1.xlsx.

**Table S2:** cam471552‐sup‐0004‐TableS2.xlsx.

**Table S3:** cam471552‐sup‐0005‐TableS3.xlsx.

**Table S4:** cam471552‐sup‐0006‐TableS4.xlsx.

## Data Availability

The data presented in this study are available from the corresponding author upon reasonable request.
