# Supplementary material for: Impact of Prior Treatment History on Recurrence After Complete Response to Atezolizumab Plus Bevacizumab in Unresectable Hepatocellular Carcinoma
Source: Cancer Med. 2026 Jan 26;15(2):e71552. doi: 10.1002/cam4.71552 (PMC12835544; doi:10.1002/cam4.71552)
Supplement: Supplementary file 1 — Figure S1: cam471552‐sup‐0001‐FigureS1.pptx. [file CAM4-15-e71552-s002.pptx]

## Slide 1
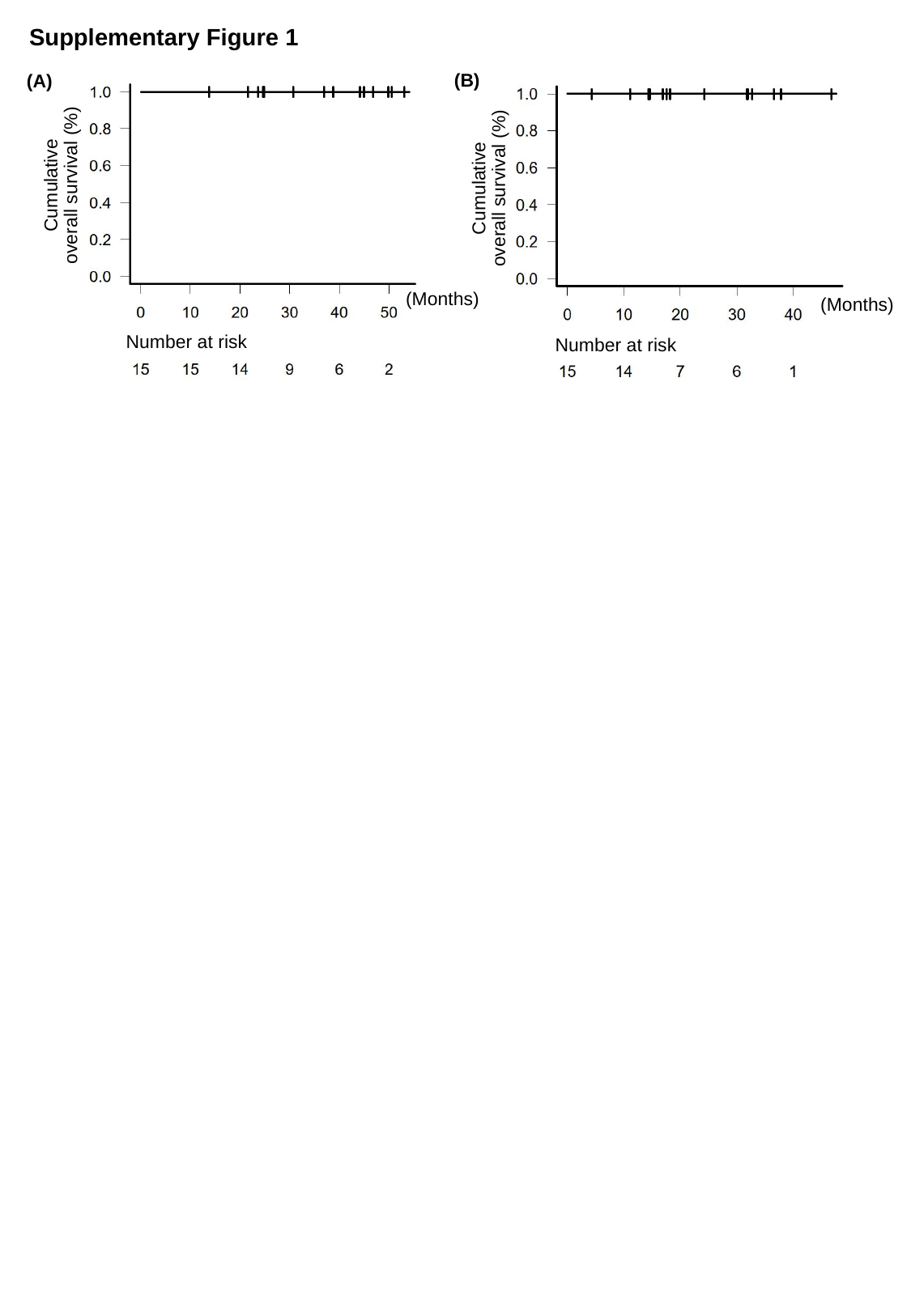

Supplementary Figure 1
(B)
(A)
Cumulative
overall survival (%)
Cumulative
overall survival (%)
(Months)
(Months)
Number at risk
Number at risk
