# Supplementary figures and images for: Impact of Prior Treatment History on Recurrence After Complete Response to Atezolizumab Plus Bevacizumab in Unresectable Hepatocellular Carcinoma
Source: Cancer Med. 2026 Jan 26;15(2):e71552. doi: 10.1002/cam4.71552 (PMC12835544; doi:10.1002/cam4.71552)

## Slide 1
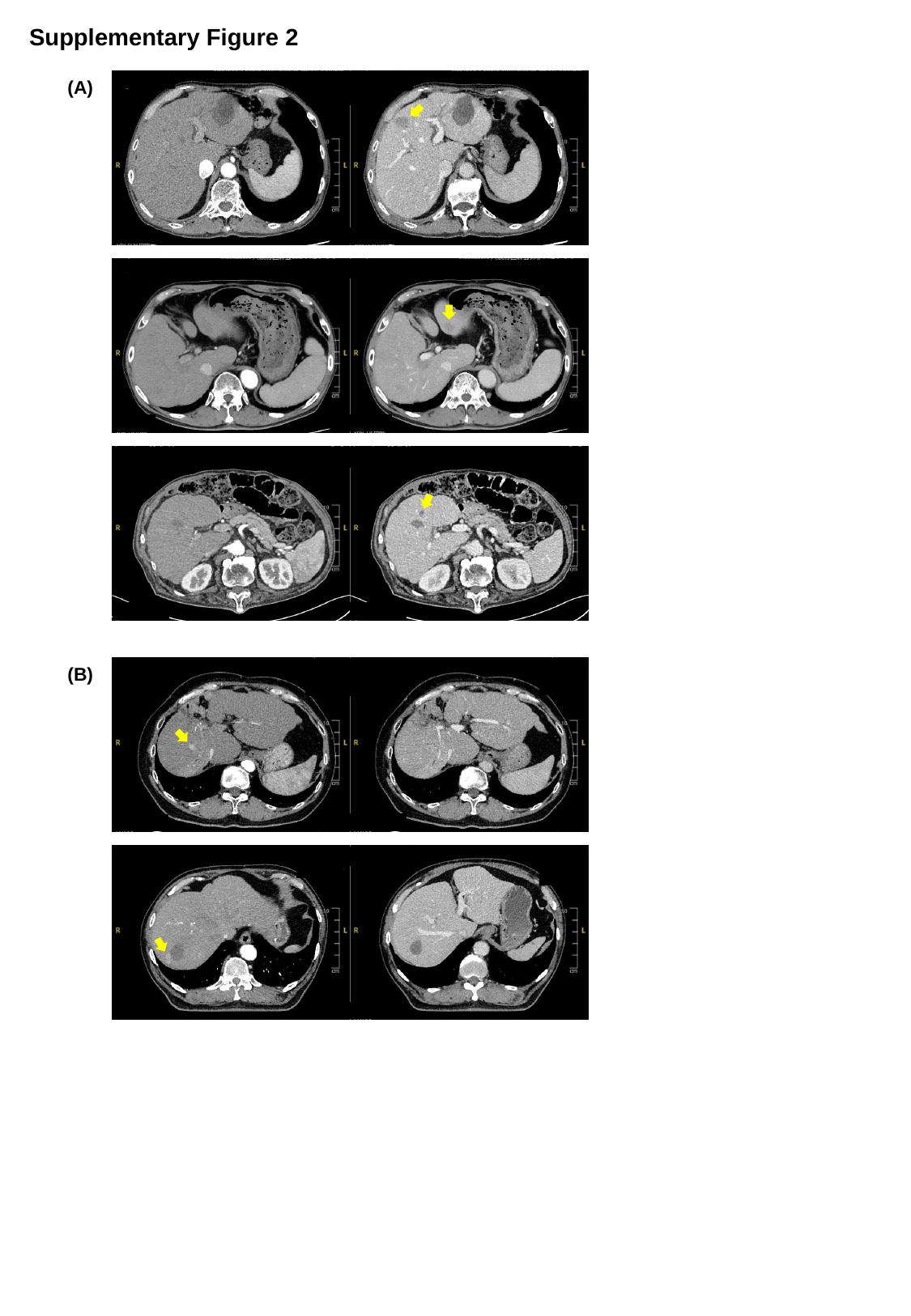

Supplementary Figure 2
(A)
(B)

Supplement: Supplementary file 2 — Figure S2: cam471552‐sup‐0002‐FigureS2.pptx. [file CAM4-15-e71552-s004.pptx]
